# The Role of Melatonin on Caprine (*Capra hircus*) Sperm Freezability: A Review

**DOI:** 10.3390/antiox13121466

**Published:** 2024-11-28

**Authors:** Alberto Jorge Cardenas-Padilla, Francisco Jimenez-Trejo, Marco Cerbon, Alfredo Medrano

**Affiliations:** 1 Laboratorio de Reproducción Animal, Unidad de Investigación Multidisciplinaria, Facultad de Estudios Superiores Cuautitlán, Universidad Nacional Autónoma de Mexico, Cuautitlán Izcalli 54714, Mexico; amedrano@unam.mx; 2 Instituto Nacional de Pediatría, Coyoacán, Mexico City 04530, Mexico; trejofjj@gmail.com; 3 Departamento de Biología, Facultad de Química, Universidad Nacional Autónoma de Mexico, Coyoacán, Mexico City 04510, Mexico; mcerbon85@yahoo.com.mx

**Keywords:** goat, melatonin, antioxidant, spermatozoa, freezability, oxidative stress

## Abstract

In mammals, the pineal hormone melatonin is the most powerful pacemaker of the master circadian clock and is responsible for reproduction in seasonal breeders. It is also well known that melatonin and its metabolites play antioxidant roles in many tissues, including reproductive cells. Melatonin synthesis and secretion from the pineal gland occurs during scotophase (the dark phase during a day–night cycle), while its inhibition is observed during photophase (period of light during a day–night cycle). Short-day breeders, such as goats, are stimulated to breed in a manner dependent on high endogenous levels of melatonin. This hormone can be synthesized in various extra-pineal tissues, such as retina, gastrointestinal tract, ovaries, and testis, with its main function being as a local antioxidant, given that melatonin and its metabolites are potent scavengers of reactive oxygen and nitrogen species. Moreover, it has been reported that some functions of melatonin can be exerted through plasma membrane and intracellular receptors expressed in the male reproductive system, including germ cells, immature and mature spermatozoa. It has been shown that melatonin may enhance gamete cryosurvival mainly by its addition into the media and/or in exogenous melatonin treatments in several species. In the present review, the physiological effects of endogenous melatonin in mammals are described, with a deeper focus on caprine reproduction. Additionally, results from recent investigations on the roles of exogenous melatonin aimed at improving the reproductive efficiency of goat bucks are discussed. There are contradictory findings and a limited amount of research available in the field of goat sperm cryopreservation associated with the use of melatonin. Understanding and improving goat reproduction and production is essential for many marginalized human populations around the world who directly depend on goats to maintain and improve their lifestyle.

## 1. Introduction

Melatonin (N-acetyl-5-methoxytryptamine; MLT) is an amphiphilic indolamine that is derived from tryptophan [1,2] and is present in all types of organisms, where it has evolved to perform multiple roles [3]. In particular, the discovery and chemical characterization of MLT opened a new area of research in the field of seasonal reproduction [4,5].

### 1.1. Biosynthesis and Secretion

The main site of MLT synthesis and secretion in vertebrates is the pineal gland, which occurs during the dark phase of the circadian pattern [6]. MLT synthesis is dependent on the enzyme N-acetyl-transferase (NAT), and the secretion pattern of this enzyme is regulated by the intensity of light perceived by the retina. MLT plays a role as a local antioxidant, as well as a regulator of the biological clock and reproductive seasonality [5]. MLT is the main regulator of reproduction in seasonal breeders such as goats (*Capra hircus*) and is the signal that either inhibits or stimulates reproductive activity in different animal species [7]. Thus, sexual behavior and semen quality vary throughout the year, declining during the non-reproductive season [8].

### 1.2. Mechanisms of Action

The antioxidant effects of MLT can be exerted by MLT itself and/or via its metabolites (produced during its degradation), having main effects at the local level, such as improving testicle and semen characteristics [9,10]. Males with high levels of endogenous MLT, as well as males receiving exogenous MLT, show higher libido and better seminal quality [11]. As an antioxidant, MLT has functions including the scavenging of free radicals, regulating reactive oxygen (ROS) and nitrogen species (NOS), preventing lipid peroxidation, and reducing oxidative cellular stress [12,13]. ROS and NOS can freely cross cell membranes, including the blood–brain and blood–testicular barriers [12].

Some actions of MLT involve specific receptors at both the plasma membrane and cell nucleus. Two different MLT membrane receptors, MT1 and MT2, have been well identified and characterized, which developed at a later stage during evolution to exert receptor-mediated biological functions of MLT rather than antioxidant functions [14,15]. These MLT receptors are widely distributed in mammalian tissues, including the brain, retina, lens, bone marrow, skin, gastrointestinal tract, ovaries, testis, and sperm [16]. Both receptors belong to the family of seven-(pass)-transmembrane receptors, also called G-protein-coupled receptors (GPCRs) [17,18,19,20,21]. Both receptors show high homology in their amino acid sequences and are linked to the inhibition of cAMP production [16,20,22]. The dissociation constant (Kd) of the sites described with MLT as a ligand is on the nanomolar order (10^−8^ to 10^−9^ M), indicating a greater affinity towards MT1 than MT2 [23]. Most of the physiological functions of MLT involve its interaction with these receptors; for example, both receptors are involved in the circadian rhythm and play an important role in the endocrine and reproductive functions of mammals [17,24,25].

There is another membrane receptor, MT3, which is considered a membrane receptor showing low affinity for MLT [26]; however, the coupling site of MT3 is still unknown, and the cascade of signal transduction as well as the biological consequences after its stimulation have not yet been discovered [20]. There is evidence that the MT3 binding site is an enzyme, quinone 2 reductase (QR2), instead of an MLT membrane receptor [27].

Although the effects of MLT have been associated with its antioxidant properties and its receptor-mediated signalling, its mechanism of action is still not entirely clear [6]. The effect of MLT on the cytoskeleton mediated by calmodulin, in addition to its effect stimulating the influx of Ca^2+^ into sperm cells, has been described as part of its mechanism of action [28].

## 2. REDOX Regulation in Spermatozoa

Spermatozoa are very specialized cells that undergo a unique process that occurs during spermatogenesis that provides them the capacity to fertilize the oocyte. The main modifications that occur during sperm formation, according to Avidor-Riess et al. [29], are as follows: (i) the manchette reshapes the nucleus, (ii) the protamine repacks the DNA, (iii) new RNA granules form, (iv) the protein-based centrioles are remodeled, (v) a flagellum with a unique configuration is formed, (vi) membrane-bound organelles such as Golgi and ER are converted and reduced to acrosome and residual bodies, and (vii) 80S ribosomes along with most of the cytoplasm are eliminated. These modifications, while sperm are formed, limit the DNA-damage detection and repair mechanisms through antioxidant defences. In addition, a high concentration of polyunsaturated fatty acids (PUFAs) contained in spermatozoa plasma membranes, essential for the membrane fluidity during fertilization, negatively affects sperm oxidation status [30].

Oxidation in cells is directly related to ROS and NOS production, which is associated with cell metabolism, with mitochondria being the major site of its formation. It has been described that sperm spontaneously produce a variety of reactive species, such as hydrogen peroxide, superoxide anion, and oxide nitrogen [31,32]. Moreover, controlled production of ROS has been associated with physiological processes and signalling pathways in the context of male fertility [33]. Sperm capacitation, hyperactivation, acrosome reaction, and sperm oocyte fusion are modulated by ROS, mainly through tyrosine phosphorylation cascades [33,34]. Capacitation also involves cholesterol remotion (cholesterol efflux) from the sperm plasma membrane, being preceded by its oxidation [32].

Spermatozoa have limited antioxidant defences that can be overwhelmed by ROS production, inducing oxidative stress [31,32,35]. This oxidative stress can be related to osmotic stress when cryopreservation is performed, inducing premature capacitation [36]. The exacerbated production of ROS—regardless of whether they were produced by the sperm freezing process—can induce lipid peroxidation in the plasma membrane and DNA fragmentation, disrupting the rate of fertilization. Membrane lipid peroxidation has been measured according to malondialdehyde (MDA) formation, while DNA fragmentation can be tested using many different techniques, with the drawback that they are not specific for oxidation-induced damage [31,35]. Oxidative stress is responsible for DNA damage in sperm by two main mechanisms: gene mutation and direct breakdown of the DNA backbone [37]; the excessive presence of ROS and NOS produces poor chromatin compaction and incomplete protamination. Also, oxidative DNA lesions in sperm impair active DNA demethylation during epigenetic reprogramming in early embryonic development [38].

The balance between ROS production and scavenging is essential for correct sperm function. The oxidative stress in spermatozoa is modulated in a dependent manner on different factors, including (i) enzymatic antioxidant defences, such as glutathione (GSH), superoxide dismutase (SOD), and catalase (CAT), and (ii) non-enzymatic defences, such as the presence of ROS scavengers like α-tocopherol (vitamin E), selenium, zinc, and MLT and its metabolites [35,39].

It has been shown that goats have higher tolerance to pollutants, such as toxic heavy metals (e.g., Pb), in comparison to cattle [40]. On the other hand, Cd has been used to induce toxicity in this species, causing a diminishing in liver levels of enzyme antioxidant activity, since the main function of the liver is to filter the blood and remove toxins [41]. In vitro assays in caprine testicular germ cells have also induced oxidative toxicity and apoptosis in spermatogenic cells [42].

## 3. Sperm Cryopreservation and Its Association with Oxidative Stress

**Figure 1:**
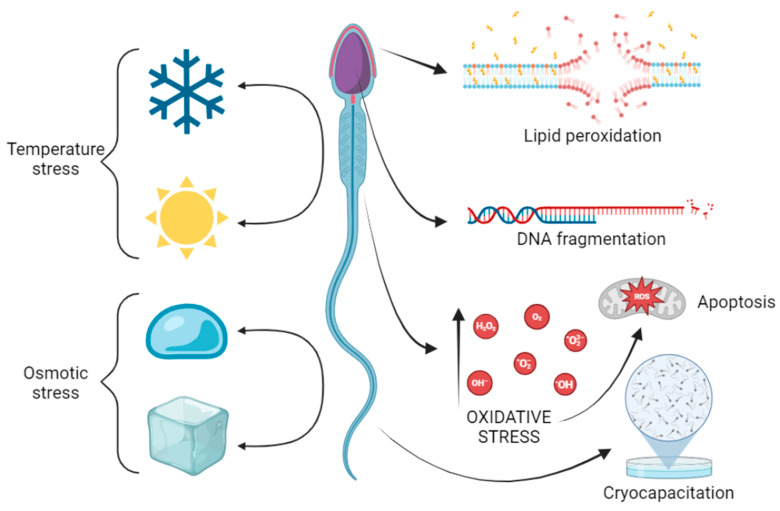
Effects of temperature, osmotic, and oxidative stresses in spermatozoa during cryopreservation.

Sperm cryopreservation is often associated with cell membrane destabilization, the loss of surface proteins, decreased motility, mitochondrial activity, and increased ROS generation [39]. Subjecting spermatozoa to freezing–thawing processes during cryopreservation involves a sequence of physical–chemical stressors that can modify the quality of sperm and its fertilizing potential. In an attempt to preserve germplasm obtained from males, Medrano et al. [43] described the main events that a sperm must face when the temperature is modified, namely, ice crystal formation, the solution effect, and ice melting. They mentioned at least three main types of stress, which are all linked to each other: (i) temperature stress, (ii) osmotic stress, and (iii) oxidative stress. The effects of these stressors on cryopreserved spermatozoa were determined after thawing, inducing a reduction in fertilizing capacity, which is also known as cryocapacitation (Figure 1).

According to Bollwein and Bittner [44], the mechanisms that increase oxidative stress in frozen–thawed sperm are not yet clear; however, it can be attributed to depletion of antioxidative enzymes or to osmotic stress during sperm freezing and thawing. McCarthy et al. [45] researched the possible association between osmotic and oxidative stress through the incubation of spermatozoa in anisosmotic, media which resulted in an increased ROS generation; also, they observed that lipid peroxidation was significantly decreased in the presence of antioxidants, such as α-tocopherol, concluding that cryopreservation-induced osmotic stress may lead to oxidative cell damage.

Cryocapacitation (premature capacitation) leads to membrane reorganization, accompanied by the loss of polyunsaturated fatty acids and cholesterol, which play important roles in maintaining the natural fluidity and integrity of the cell membrane right after thawing, thus negatively impacting the rate of fertilization [39].

## 4. Effects of MLT on Mammal Spermatozoa

Seasonal variations in MLT in the blood plasma of seasonal species, such as rams and goats, seem to be partially responsible for the differences in fertility and semen quality between reproductive and non-reproductive seasons. These effects have also been observed in males receiving exogenous MLT [46,47,48] and in males with high levels of MLT present in seminal plasma [49].

The presence of MLT in seminal plasma has been demonstrated in different species, including humans and rams. In humans, the presence of MLT in seminal plasma has been demonstrated [50], as well as the expression of MLT receptors in spermatozoa [51], suggesting a direct action of MLT on the sperm cell. High levels of endogenous MLT correlate with improved semen quality; furthermore, the addition of MLT to semen improved sperm motility [11]. It has been reported that exogenous MLT stimulates testicle development and improves semen quality in rams during the non-reproductive season [7,52]. Exogenous MLT, given to rams during the non-reproductive season, may compensate for the negative effects of low levels of both GnRH and LH on sexual behavior, testicle parameters, spermatogenesis, and fertility [49].

MLT added to different media, in the presence or absence of hydrogen peroxide, has improved pig sperm quality in terms of motility, viability, plasma membrane integrity, and mitochondrial activity when compared to control groups [53]. Furthermore, supplementation of freezing medium with MLT improved bull sperm motility, viability, normal morphology, and plasma membrane integrity after thawing [54]; these authors claimed that MLT reduced lipid peroxidation and increased both enzymatic and total antioxidant activity.

In vitro treatment of mouse sperm reduced oxidative cellular damage and levels of ROS and NOS [55], peroxidation of membrane lipids [56,57], apoptosis markers [58,59], and DNA fragmentation [60]. Similarly, exposure of rat sperm to MLT prevented damage induced by oxidative stress, avoiding decreases in both enzymatic antioxidant activity and testosterone levels [61,62], reducing sperm abnormalities [63], protecting sperm chromatin from decondensation, and promoting the production of functional sperm [62].

On the other hand, MLT participates in capacitation and acrosome reactions, processes that involve plasma membrane reorganization, including the internalization of MLT receptors [64]—a mechanism of receptor desensitization already demonstrated for MT1 in humans [65], mice [66], and rams [67] after a brief exposure to MLT.

There are very few reports on the use of MLT in goat sperm. Administration of MLT to goat bucks significantly improved libido and sperm quality—namely, wave motion, progressive motility, and viability—soon after the insertion of subcutaneous MLT implants [68].

The main effects of MLT reported previously have been linked to different pathways of action and were in-depth discussed by Alevra et al. [35]. It has been shown that MLT can (i) reduce the excessive production of free radicals; (ii) upregulate the expression of HSP90 (heat shock protein 90); (iii) upregulate antioxidant enzymes and enhance their functionality; (iv) regulate MPTPs (mitochondrial permeability transition pores); (v) reduce lipid peroxidation; (vi) stabilize membrane integrity; (vii) prevent the leakage of intracellular enzymes; and (viii) act as an anti-apoptotic molecule.

Our research group has tested different MLT concentrations added to freezing diluent in several species in order to determine the amount that improves the quality of post-thawed sperm (Table 1). We have found that the species, breed, and season are some factors that can modify the sperm response to MLT in vitro treatments; the results obtained are not conclusive but are not discouraging either.

### MLT Receptors in Spermatozoa

MT1 and MT2 receptors have been identified in the ram sperm plasma membrane [76], and it has been suggested that the effect of MLT on ram sperm capacitation may be mediated by these receptors [49,77]; however, the biochemical pathway is still unknown. MLT may decapacitate previously capacitated sperm through MT2; incubation of sperm with MT2 antagonists increased the rate of capacitated sperm, while incubation with agonists under capacitating conditions maintained the proportion of non-capacitated sperm [39].

The distribution of MT2 in the ram plasma membrane seems to be related to sperm capacitation status [77], as a strong correlation has been found between the pattern of intracellular calcium distribution and the signal intensity of MT2 via indirect immunofluorescence. Both bicarbonate and calcium are key actors that initiate capacitation and acrosome reactions in ram sperm [78,79]. In addition, a modulatory effect of MLT on ram sperm functionality during in vitro capacitation has been observed, mediated mainly by the MT2 receptor [24].

MLT and its receptors have been detected in seminal plasma and the sperm plasma membrane of donkeys, stallions, boars, bulls, and dogs [25]. It has been shown that MLT protects rabbit spermatozoa from cryo-damage via decreasing oxidative stress, improving sperm motility, membrane integrity, acrosome integrity, and mitochondrial membrane potential, as well as AMP-activated protein kinase (AMPK) phosphorylation [80,81]. In dogs, MLT improved sperm cryosurvival through decreasing the percentage of hyper-fluid membranes, and reduced damage to the acrosome and sperm associated with acrosomal reactions after thawing [70]. In buffalo, MLT improved seminal quality parameters including viability, total motility, progressive motility, linear velocity, and decreasing sperm morphological abnormalities [82].

## 5. MLT and Goat Reproduction

### 5.1. Endogenous Variation in MLT Levels in Goats

Alila-Johansson et al. [83,84] carried out a couple of investigations focused on the MLT rhythm in Finnish Landrace goats. The first study [83] showed that, as in other species, the MLT signal carries reliable information about seasonal changes in photoperiod in goats. Some information was conserved in the endogenous rhythmicity of MLT secretion, at least for one day in constant darkness. The long-day signal was better conserved in late spring than early fall, suggesting that endogenous MLT secretion can be modulated by circannual clock mechanisms and/or long-term photoperiodic history. Their results suggested that, in addition to the evident light-adjusted melatonin rhythm, the endogenous rhythm of melatonin secretion varies throughout the year. In their second study, Alila-Johansson et al. [84] demonstrated that the MLT rhythm was probably dominated by the evening oscillator as, in all individuals and in all seasons, MLT levels increased immediately after the light offset. There was inter-individual variation in the timing of MLT decline, and, in winter, high levels of MLT were not maintained over the whole dark period in any individual animal. According to their results, goats must have effective intra- or extra-pineal feedback mechanisms that arrest MLT synthesis, depending on its start time and independent of the continuing darkness.

To evaluate the influence of the natural photoperiod on the daily rhythm of MLT in goats, Carcangiu et al. [85] assessed MLT plasma concentrations at four different times of the year. They collected samples from Sarda goats by means of a cannula inserted into the jugular vein every two hours for a 24 h period during the following moments of the year: vernal equinox, summer solstice, autumn equinox, and winter solstice. They observed the existence of a clear seasonal variation in the daily rhythm of plasma MLT in goats, with the highest midline estimating statistic of rhythm (MESOR) value in winter followed by spring. Their results showed a daily rhythm of plasma MLT concentration strongly related to the seasons.

Farsi et al. [86] investigated MLT plasma variations in desert goats under total darkness (DD) conditions, and they found that plasma MLT follows an ambient temperature (Ta) cycle, with its peak occurring only in the cryophase (low temperature during a 24 h cycle). They concluded that a Ta cycle, and not just scotophase in the circadian rhythm, is the true “zeitgeber” in goats that can modify the MLT concentration.

Our research group [87] observed no differences in MLT levels in the seminal plasma of goat bucks between breeding and non-breeding seasons, which may be due to the latitude (19°69′) at which the bucks were kept. The MLT levels found in our research showed a higher concentration in seminal plasma (521.87 pg/mL) in comparison to other species like rams (200 pg/mL) [8]. However, we found that the intra-seasonal variation in MLT was lower in reproductive than non-reproductive seasons, likely responding more to ambient temperature than to circadian rhythmicity [86]. Moreover, we identified some variation in sperm quality throughout the year, which could be associated with certain “zeitgeber” factors such as temperature variation and photoperiod.

### 5.2. Differences in MLT Concentration Between Male and Female Goats

To the best of our knowledge, only one study has reported differences in the MLT concentrations in plasma between male and female goats. Kaushalendra [88] measured the MLT concentration in plasma from female goats, castrated male goats, and intact male goats of the Jamunapari breed, sampling during scotophase throughout the year. Higher MLT concentrations were found in females (295.7 ± 1.05 pg/mL) and castrated males (197.58 ± 1.56 pg/mL) than in intact males (151.88 ± 1.30 pg/mL). In all groups, an annual variation in MLT concentration was observed, increasing from August onwards until November, and the lowest concentrations of MLT were recorded from March to July (summer season). In intact males, testicular weight showed a mild correlation with plasma MLT concentration, suggesting that MLT might have a limited or no role in the regulation of reproductive functions in this species, according to the author.

### 5.3. Sperm Quality and Its Relationship with MLT in Goat Bucks

Zarazaga et al. [89] modified the photoperiod of Mediterranean goat bucks (long days) and inserted MLT implants during the non-reproductive season. Treated groups showed higher concentrations of plasma testosterone and semen volume than the control group.

On the other hand, Vince et al. [68] assessed the effects of exogenous MLT on libido and semen quality parameters during the non-breeding season. Their results indicated that the application of MLT did not modify libido intensity. However, it improved qualitative parameters of semen in goat bucks, such as wave motion, progressive motility, and the proportion of live spermatozoa shortly after the insertion of MLT implants.

Samir et al. [90] investigated the potential role of exogenous MLT on testicular blood flow, circulating hormones, and semen characteristics in Shiba goats. They found that MLT increased sperm quality (wave motion, progressive motility, and the proportion of live sperm) in the treatment group (single s.c. dose of 36 mg/goat), in comparison to the control group. Furthermore, they observed an increase in testicular blood flow; thus, MLT seems to improve the fertility of male goats.

Tölü et al. [91] studied MLT and testosterone hormone levels, as well as mating behaviors, in Turkish Saanen goat bucks after the application of MLT implants during the non-breeding season. They found that the implant caused significant increases in MLT (526.3 ng/L vs. 199.0 ng/L) and testosterone (12.7 nmol/L vs. 6.13 nmol/L) levels, as well as positive effects on the frequency of mating behavior.

### 5.4. Sperm Freezability and Its Relationship with MLT in Goat Bucks

MLT has been used as an additive in sperm freezing diluents, producing good results in terms of sperm cryosurvival in long- and short-day breeders (horses and rams, respectively) and even in non-seasonal breeders (humans and pig); this suggests a direct action of MLT on the hypothalamus–hypophysis–testicles axis [20].

In the case of goat buck preserved sperm, some contrasting results have been reported. Moazzami et al. [92] found that the quality of goat buck spermatozoa was similar without or with MLT (0.0, 1.0, or 2.0 mM) added to the freezing medium, considering 2 days of storage at 5 °C. However, a negative effect of DMSO (employed to dissolve MLT) on sperm quality was detected. On the other hand, Gallego-Calvo et al. [93], who worked with Blanca Andaluza bucks, showed that exogenous MLT (subcutaneous MLT implants) improved fresh sperm motility characteristics in comparison to those recorded for two months of either short (8 h of light/dark) or long day (16 h of light/dark) regimes. However, no significant differences were observed in the motility of frozen–thawed spermatozoa between treatments (MLT, short days, and long days). Moreover, El-Battawy [94] tested the influence of MLT on sperm motility of Zaraibi goat sperm stored at 5 °C for seven days. Their results showed that MLT—particularly at the highest concentration (20.0 vs. 0.0, 10.0, and 15.0 µg)—significantly improved sperm motility, reduced dead sperm, and improved post-thaw sperm motility. Furthermore, Tanhaei-Vash et al. [95] tested the effects of MLT, myo-inositol, and a combination of both on buck sperm cryosurvival. After freeze–thawing, sperm motility, viability, plasma membrane, and acrosome intactness improved significantly in the MLT and myo-inositol groups in comparison to the control group. However, co-supplementation improved the same parameters in comparison to MLT or myo-inositol alone, indicating a synergistic effect at 5 µM myo-inositol and 1 mM MLT.

On the other hand, Monteiro et al. [96] supplemented MLT in freezing skimmed milk or Tris-egg yolk-based extenders for Saanen buck ejaculates; they found that MLT did not improve post-thawing sperm quality and that the high concentration treatment (4.0 vs. 0.0, 0.5, 1.0, or 2.0 mM) may be deleterious. Furthermore, our research group [75] supplemented MLT in Tris-egg yolk-based freezing extender in Saanen buck semen and observed no differences between groups (0.0, 1.5, or 3.5 mM); notably, MLT did not improve sperm cryosurvival.

**Figure 2:**
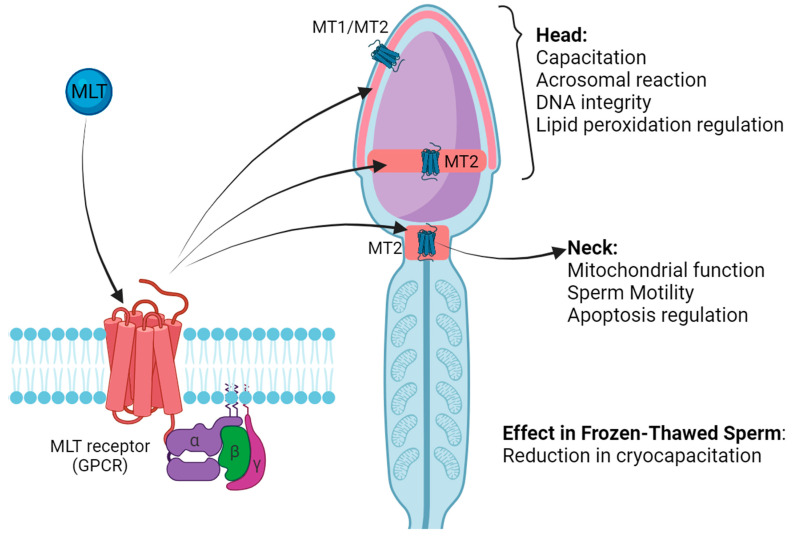
Topographic localisation of MLT receptors in goat buck sperm plasma membrane and their possible effects on sperm physiology.

Our recent investigation [87] showed that MLT receptor expression in goat buck sperm plasma membranes varies throughout the year. Western blot analysis revealed a band for MT1 (MW: 16 kDa) and multiple bands for MT2 (MW: 28, 35, 42, and 75 kDa), while the densitometry analysis revealed that the relative expression of the bands differed between breeding and non-breeding seasons (except the 75 kDa band), being higher in winter than in summer. These seasonal variations in the expression of receptors were associated with capacitation status when performing a standard cryopreservation protocol using a Tris-egg yolk-based diluent. We also showed that the topographical localization of MLT receptors differs when comparing MT1 vs. MT2 receptors in the goat sperm plasma membrane (MT1 was localized mainly in the apical region of the head, while MT2 was localized mainly in the neck as well as the apical region and equatorial segment of the head). These findings suggest that each receptor could be involved in sperm quality and freezability modulation and/or regulation through different signalling pathways, in addition to the associated antioxidant properties (Figure 2).

Budiyanto et al. [97] published a meta-analysis focused on the use of exogenous MLT and its effects on sperm quality in small ruminants. The authors included a total of 30 research articles, of which only 6 (20%) included goats in their investigations. This meta-analysis only included research in which MLT was not used for in vitro treatments. The number of papers seems to be not so small, in comparison with rams; however, the investigations were published over a 24-year period (between 2001 and 2024), averaging one paper published every 4 years, worldwide. This means that research in goats is still lagging when compared to other species.

## 6. Conclusions and Future Directions

Recent research on the link between MLT and reproduction in goat bucks suggests that it could differ from that in other seasonal breeders, such as rams. The role of endogenous and exogenous MLT on caprine physiology is still unclear, even when most investigations in the existing literature have revealed some positive effects of MLT in the context of sperm quality and freezability. Accordingly, it is necessary to further investigate the physiology of goat buck reproduction regarding the role of MLT and its receptors. It should be considered that sheep and goats do not have identical physiology, even if they are both short-day seasonal breeders. The results presented in this review demonstrate that the use of MLT in different protocols for caprine sperm cryopreservation is paradoxical and inconclusive.

The presence of MLT in seminal plasma and MLT receptors in the sperm plasma membrane has been reported in many species and more recently in goat bucks by our group. As the expression of MT1 and MT2 could be correlated to sperm quality, researchers may use different approaches for freeze–thawing of caprine spermatozoa, thus improving reproductive technologies. Further investigation is required to determine whether endogenous levels and variations in MLT in goat buck seminal plasma occur at higher latitudes and the specific effects of MLT receptor expression in the sperm plasma membrane.

At present, different research groups are investigating the effects of several antioxidants and their impacts on sperm. MLT and its physiological participation have been described in some species, but its role in the prevention of premature capacitation when a cryopreservation protocol is performed in goat buck spermatozoa remains unclear. We have standardized a new approach for describing the cholesterol efflux in spermatozoa from goats [98]; with these results, we hope to obtain more data regarding the effects and function of MLT in the prevention of cryocapacitation when using a standard freezing protocol.

Considering that the reproductive pattern of goats is seasonal, its products (meat and milk) are only available for a limited part of the year; thus, it would be convenient to develop techniques allowing for continuous reproduction. Assisted reproduction techniques applied to goats, both in females and males, should be considered as an option. This implies that researchers should invest their efforts in improving the knowledge through investigation in any field of caprine production because, for the most underprivileged strata of society worldwide, goats are often the only source of income and animal protein. The antioxidant effects of MLT have been applied in semen cryopreservation, but its effects may also be considered in the context of embryo production.

## Figures and Tables

**Table 1:** Effects of melatonin addition to freezing diluents on sperm post-thaw quality (data from our research group).

Species	MLT Treatment Groups (mM)	Effects on Post-Thawed Sperm Quality	Author
Dog	0.0 1.0 2.0	There were no differences in sperm quality between MLT treatments (*p* > 0.05)	[69]
	0.0 0.5 2.0 3.5	High MLT dose groups (2.0 and 3.5) showed improved sperm cryosurvival: Increase in intact acrosome and capacitated acrosome-intact, and decrease in hyper-fluid membranes and acrosome-reacted (*p* < 0.05)	[70]
	0.0 3.5 3.75 4.0 4.25	There were no differences in sperm quality between MLT treatments (*p* > 0.05)	[71]
Boar	0.0 1.0 2.0	There were no differences in sperm quality between MLT treatments (*p* > 0.05)	[72]
	0.0 3.0 5.0	There were no differences in sperm quality between MLT treatments (*p* > 0.05)	[73]
Rabbit	0.0 0.1 0.75 1.5 2.25 3.0	There were no differences in sperm quality between MLT treatments (*p* > 0.05)	[74]
Goat	0.0 1.5 3.5	Wave motion was increased in both MLT treatment groups vs. control group (*p* < 0.05)	[75]

## Data Availability

No new data were created or analyzed in this study. Data sharing is not applicable to this article.
